# MiR-942-5p inhibits tumor migration and invasion through targeting CST1 in esophageal squamous cell carcinoma

**DOI:** 10.1371/journal.pone.0277006

**Published:** 2023-02-27

**Authors:** Liangming Zhang, Sunxing Yu, Xiaoqing Yin, Mingshu Tu, Liqing Cai, Yi Zhang, Lili Yu, Songgao Zhang, Xiaojie Pan, Yi Huang

**Affiliations:** 1 Provincial Clinical College, Fujian Medical University, Fuzhou, China; 2 Department of Clinical Laboratory, Fujian Provincial Hospital, Fuzhou, China; 3 Health Management Center (Preventive Treatment), the Second Affiliated Hospital of Fujian University of Traditional Chinese Medicine, Fuzhou, China; 4 Integrated Chinese and Western Medicine College, Fujian University of Traditional Chinese Medicine, Fuzhou, China; 5 Department of Thoracic Surgery, Fujian Provincial Hospital, Fuzhou, China; 6 Central Laboratory, Fujian Provincial Hospital, Fuzhou, China; 7 Center for Experimental Research in Clinical Medicine, Fujian Provincial Hospital, Fuzhou, China; 8 Fujian Provincial Key Laboratory of Critical Care Medicine, Fuzhou, China; Chung Shan Medical University, TAIWAN

## Abstract

**Introduction:**

Cysteine Protease Inhibitor 1 (CST1), a cystatin superfamily protein with the effect on the inhibition of cysteine protease activity, is reported to be involved in the development of many malignancies. MiR-942-5p has been demonstrated its regulatory effects on some malignancies. However, the roles of CST1 and miR-942-5p on esophageal squamous cell carcinoma (ESCC) are still unknown up to now.

**Methods:**

The expression of CST1 in ESCC tissues was analyzed by TCGA database, immunohistochemistry, and RT-qPCR, respectively. Matrigel-uncoated or-coated transwell assay was used to determine the effect of CST1 on migration and invasion of ESCC cells. Regulatory effect of miR-942-5p on CST1 was detected by dual luciferase assay.

**Results:**

CST1 was ectopically highly expressed in ESCC tissues, and had the effect on promoting the migration and invasion of ESCC cells by upregulating phosphorylated levels of key effectors including MEK1/2, ERK1/2, and CREB in MEK/ERK/CREB pathway. Dual-luciferase assay results showed that miR-942-5p had a regulatory effect on targeting CST1.

**Conclusions:**

CST1 plays a carcinogenic role on ESCC, and miR-942-5p can regulate the migration and invasion of ESCC cells by targeting CST1 to downregulate MEK/ERK/CREB signaling pathway, suggesting that miR-942-5p/CST1 axis might be a promising target for diagnosis and treatment of ESCC.

## Introduction

Esophageal cancer is a common malignant tumor of the digestive tract originating from the epithelial tissue of the esophagus and can be classified into Esophageal squamous cell carcinoma (ESCC) and Esophageal adenocarcinoma (EAC) [1]. According to 2020 global cancer burden data, esophageal cancer ranks the eighth and sixth in incidence and mortality of malignancies, respectively [2]. In recent years, the incidence rate of ESCC is significantly higher than that of EAC, and as a highly aggressive tumor, ESCC exhibits highly malignant biological behavior and leads to a dismal 5-year survival rate up to 5–20% [3]. Unfortunately, China has the highest incidence of ESCC with up to 53.3% of new cases and 55.6% of deaths in the world [2]. Therefore, it is essential to further explore the mechanism of ESCC development and define new molecular targets related to ESCC for improving the survival rate and prognosis of ESCC patients.

Cysteine proteinase inhibitor 1 (CST1) is a secreted protein belonging to the cysteine proteinase inhibitor (CST) type 2 subfamily. Aberrant CST1 expression has been reported to be associated with the occurrence, proliferation, invasion, and recurrence of several malignancies, including breast, pancrea, lung, hepatocellular carcinomas and gastric cancer [4–10]. Additionally, Yoneda et al [11] found that serum CST1 detection might be valuable for the diagnosis of colorectal cancer, presented by the significantly elevated level of serum CST1 in cancer patients, with a sensitivity of 27.7% (95.0% specificity); furthermore, the combination of serum CST1 with carcinoembryonic antigen (CEA) and glycoantigen 19–9 (CA19-9) further improved the diagnostic sensitivity to 62.9% on the basis of the guaranted specificity at 90.0%. In a pilot study, we found that CST1 was ectopically highly expressed in sera and cancerous tissues of the patients with ESCC, and serum CST1 detection contributed to the early diagnosis of ESCC [12], suggesting that CST1 might be involved in the occurrence and development of ESCC. Therefore, whether CST1 plays a potential carcinogenic role on ESCC is worth exploring.

It is well known that MicroRNAs (miRNAs) present an important regulatory role on malignancies. As a small fragment RNA, miRNAs mainly bind targeted mRNAs through 3’-UTR, leading to mRNA degradation or translation inhibition, of which miR-942-5p has been demonstrated its regulatory effects on some malignancies [13, 14]. For example, Li et al [15] reported that miR-942-5p could target DLG2 to inhibit malignant biological behaviors such as migration and invasion of colorectal cancer cells; a study by Chen et al [16] suggested that miR-942-5p might be an independent prognostic factor for patients with hepatocellular carcinoma by bioinformatics analysis. However, the role of miR-942-5p on ESCC has not yet been reported up to now.

In the present study, we found the regulatory effect of miR-942-5p on targeting CST1 and revealed the fact that the miR-942-5p/CST1 axis affects the migration and invasion of ESCC cells by regulating the downstream MEK/ERK/CREB pathway, which might provide a new promising target for diagnosis and treatment of ESCC.

## Materials and methods

### Cell culture and specimens

Human ESCC cell lines TE-1, TE-13, and KYSE150 were purchased from the Shanghai Chinese Academy of Sciences. ECA109 and 293T was purchased from Pronox Life Sciences Company. The cells were cultured in a cell culture incubator at 37°C with 5% CO_2_ saturation humidity. 67 pairs of cancer tissues (T) and paracancerous tissues (N) from patients with ESCC who were treated in Fujian Provincial Hospital from January 2021 to September 2021 were collected and tested. The study was approved by the Ethics Committee of Fujian Provincial Hospital. The ethics committee has approved the waiver of subjects’ informed consent.

### Immunohistochemistry

The dewaxed and hydrated tissue sections were repaired and stained according to the kit instructions (Fuzhou Maixin Biotechnology Limited Company, China). Then CST1 primary antibody (1:100, Abcam, Britain) was added dropwise to the tissue and incubated at 4°C overnight. The tissues were then incubated with biotinylated secondary antibody dropwise for 10 min at room temperature, followed by treatment with streptavidin-horseradish peroxidase solution, diaminobenzidine (DAB Kit, Lab Vision), and hematoxylin, and finally, the tissues were dehydrated dried, and photographed microscopically. The staining intensity was graded as 0 = undetectable, 1+ = weak staining, 2+ = moderate staining, and 3+ = strong staining.

### Vector transfection

CST1 overexpression / knockdown lentivirus was designed and constructed by the Fuzhou Carrier Biotechnology Company, China. The overexpression vector was pCDH-CMV-MCS-3xflag-EF1-mCherry-T2A-Puro; the shRNA vector was hU6-MCS-CMV-mCherry-PGK-Puro. The lentivirus was transfected into ESCC cells by Lipofectamine 3000 Reagent (Invitrogen, America), and transfection efficiency was detected by RT- qPCR and western blot.

### Real-time PCR

NucleoZOL (Macherey-Nagel, Germany) was used to extract total RNA from ESCC tissues and cells, and 1 μg of extracted total RNA was reversely transcribed into cDNA using the Reverse Transcription PrimeScript™ RT reagent Kit (Takara, Japan)/.miRcute Enhanced miRNA cDNA First Strand Synthesis Kit (Beijing Tiangen Biochemical Technology Company, China). The miRcute enhanced miRNA fluorescence quantification kit (Beijing Tiangen Biochemical Technology Company, China) was used to detect miR-942-5p levels. The TB Green Premix Ex Taq Kit (Takara, Japan) was used to detect CST1 mRNA levels. CST1、miR-942-5p、GAPDH and U6 primers were purchased from the Fuzhou Shangya Biotechnology Company, China. The sequences of CST1 primers were CST1-F:5’-GAGGAGACCATGGCCCAGTATC-3’;CST1-R:5’-AGGTCTGCGTTATAGATGCCA-3’. Hsa-miR-942-5p-F:CCGTCTTCTCTGTTTTGGCCATGTGR: (Hsa-miR-942-5p-R:Fluorescence quantitative assay kits are self-provided).

### Western blot

Total proteins were extracted according to the whole protein extraction kit (Solarbio, Beijing, China) and protein concentrations were measured using the BCA protein assay kit (Solarbio, Beijing, China). Next, total proteins were separated by SDS/PAGE electrophoresis technique and then transferred to PVDF membrane (EpiZyme, Shanghai, China), closed at room temperature for 1 h. Primary antibody was incubated overnight at 4°C, then goat anti-rabbit IgG (Beyotime, Shanghai, China) was treated at room temperature for 1 h. Finally, the proteins were developed using a developing solution (Thermo Fisher Scientific, America). The following primary antibodies were used: CST1 (1:1000,CST, America), MEK1/2 (1:1000, CST, America), p-MEK1/2(Ser217/221)(1:1000, CST, America), p44/42 MAPK (Erk1/2) (1:1000, CST, America), p-p44/42 MAPK (Erk1/2) (1:1000, CST, America), CREB (1:1000, Affinity Biosciences, America), p-CREB(ser133)(1:1000, Affinity Biosciences, America), ELK1(1:1000, Affinity Biosciences, America), p-ELK1(ser383)(1:1000, Affinity Biosciences, America), E-cadherin (1:1000, CST, America), MMP2 (1:1000, CST, America), Slug (1:1000, CST, America), and β-actin (1:1000, Beyotime, Shanghai, China).

### Dual-luciferase reporter gene assay

The plasmids including CST1-WT, CST1-MUT, pSicheck2.0 null plasmid, and four miRNAs including miR-942-5p, miR-302f, miR-92a-3p, and miR-139-5p were purchased from Fuzhou Carrier Biology Company, China. Lipofectamine 3000 Reagent (Invitrogen, America) and Dual-Luciferase® Reporter Assay System Kit (Promega, America) were used to transfer the mimics/inhibitor into 293T cells and to measure the dual -luciferase activity.

### Transwell experiments

No matrigel matrix (Corning, America)/matrigel was added in transwell chambers (Corning, America) to determine cell migration/invasion ability. In 24-well culture plates, 100 μL of medium with a low concentration of FBS with 10×10^4^ cells was added to the upper chamber of transwell and 700 μL of medium with a high concentration of FBS was added to the lower chamber and left for 24–48 h. The number of cells passing on the transwell was randomly observed after 4% paraformaldehyde fixation and crystalline violet staining.

### Statistical analysis

All experiments were repeated 3 times independently. Graphpad Prism V8 software was used for statistical analysis and charting of experimental data. Data were described as mean ± standard deviation (x±s), t-test was used to compare the differences between two groups, and fisher precision test was used to compare the positivity rates. The expression of CST1 in esophageal cancer in the TCGA database was analyzed using the UALCAN website(http://ualcan.path.uab.edu/cgi- bin/ualcan-res.p), and all differences were considered statistically significant at *P*<0.05.

## Results

### CST1 expression was upregulated in ESCC tissues

**Fig 1:**
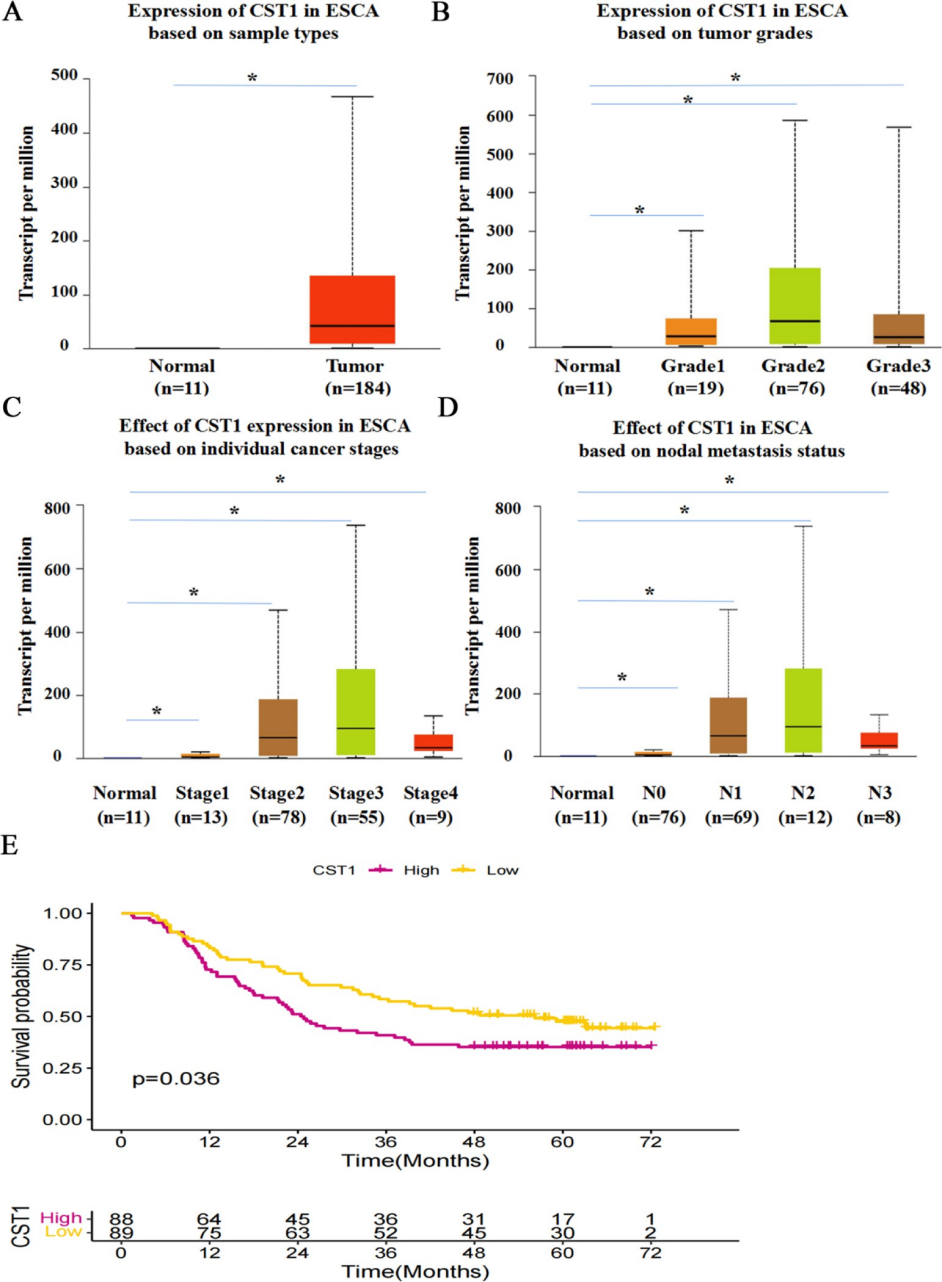
Expression of CST1 in esophageal cancer by TCGA. (A) Expression of CST1 in patients with esophageal cancer and normal subjects. (B) Expression of CST1 in patients with all grades of esophageal cancer. (C) Expression of CST1 in patients with all stages of esophageal cancer. (D) Expression of CST1 in patients with different lymph node metastases of esophageal cancer. (E) Survival probability of esophageal cancer patients with different levels of CST1 expression. (* *P*<0.05).

**Fig 2:**
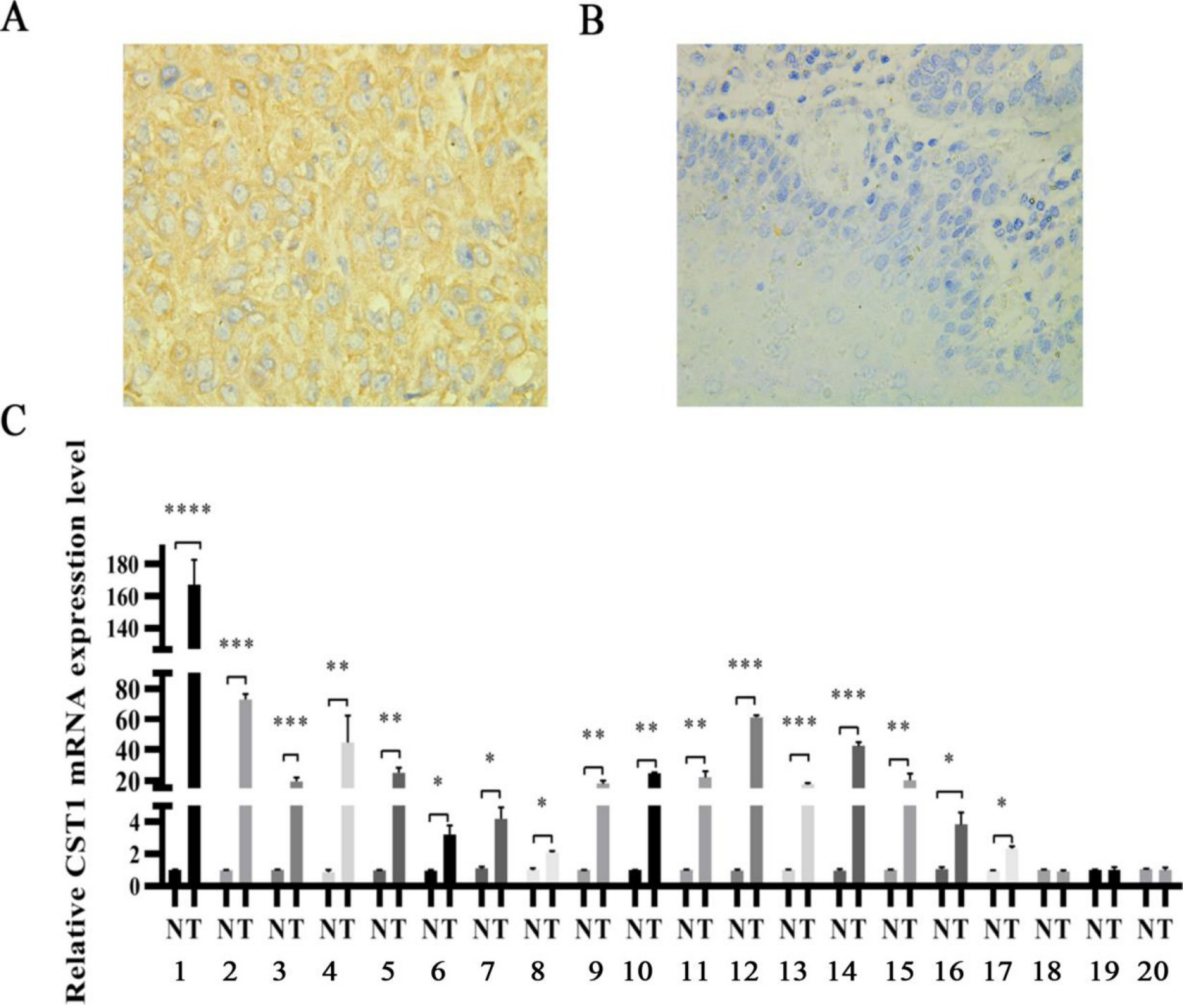
The expression of CST1 in cancerous and paired paracancerous tissues of ESCC patients (A) Positive expression of CST1 in ESCC cancer tissues (×400). (B) Negative expression of CST1 in paracancerous tissues (×400). (C) Relative expression of CST1 mRNA in 20 pairs of cancer tissues and paracancerous tissues. (* *P*<0.05,** *P*<0.01, *** *P*<0.001, ****P<0.0001).

To investigate the potential carcinogenic role of CST1 on ESCC, we first determined the expression of CST1 in ESCC tissues. By analyzing the TCGA database data through the UALCAN website, we found that CST1 expression was significantly upregulated in esophageal cancer tissues compared with normal tissues (Fig 1A–1D), and closely correlated with grades (Fig 1B), stages (Fig 1C), and lymph node metastases (Fig 1D) of cancerous tissues. In addition, we also found that the seven-year survival probability of patients with high CST1 expression in ESCC was lower than that of patients with low CST1 expression (Fig 1E). By immunohistochemical staining of 47 pairs of ESCC tissues and paracancerous tissues, CST1 protein was shown aberrant expression evidented by the positive rate up to 68.1% in cancerous tissues significantly higher than that of paired paracancerous tissues (Table 1, Fig 2A and 2B), and correlated with tumor stages; in addition, by RT-qPCR of 20 pairs of ESCC tissues and paracancerous tissues, CST1 mRNA levels in ESCC tissues were also shown significantly upregulated (Fig 2C). These results indicated that CST1 expression was upregulated in ESCC tissues and might be responsible for poor clinical performance.

**Table 1:** Positive expression rate of CST1 in cancerous and paired paracancerous tissues of ESCC patients.

Variable	n	- +		++	+++	Positivity rate (%)	*P*
Cancer	47	15	14	7	11	68.1(32/47)	<0.01
Paracancer	47	47	0	0	0	0.0(0/47)	
Stage							
I + II	26	12	6	4	4	53.8(14/26)	0.03
III + IV	21	3	8	3	7	85.7 (18/21)	

### CST1 promotes the migration and invasion of ESCC cells

**Fig 3:**
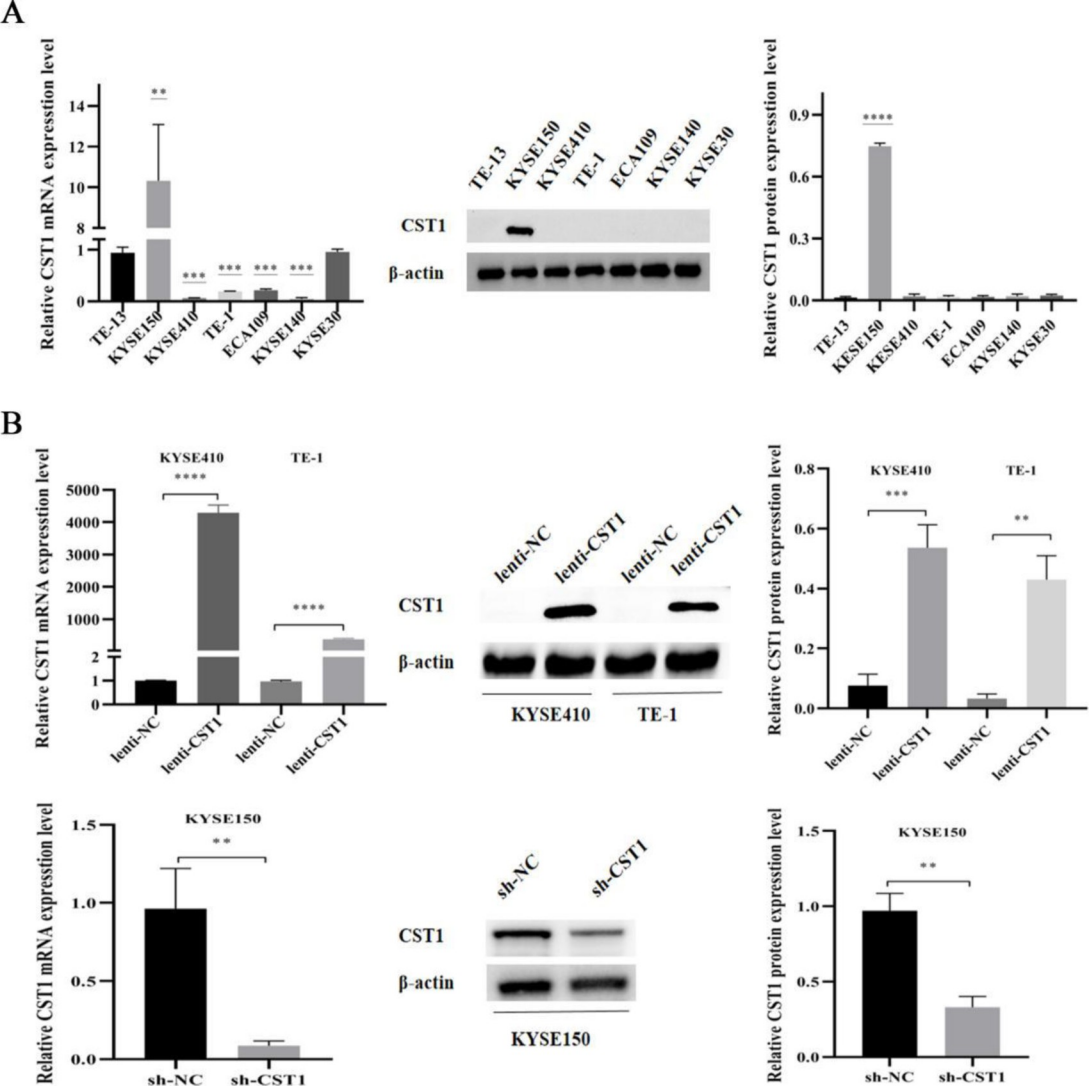
Construction of CST1 overexpression/knockdown cells. (A) CST1 mRNA and protein expression in 7 cell lines (vs TE-13). (B) mRNA and protein expression of CST1 overexpression/knockdown cells. (** *P*<0.01, *** *P*<0.001, ****P<0.0001).

**Fig 4:**
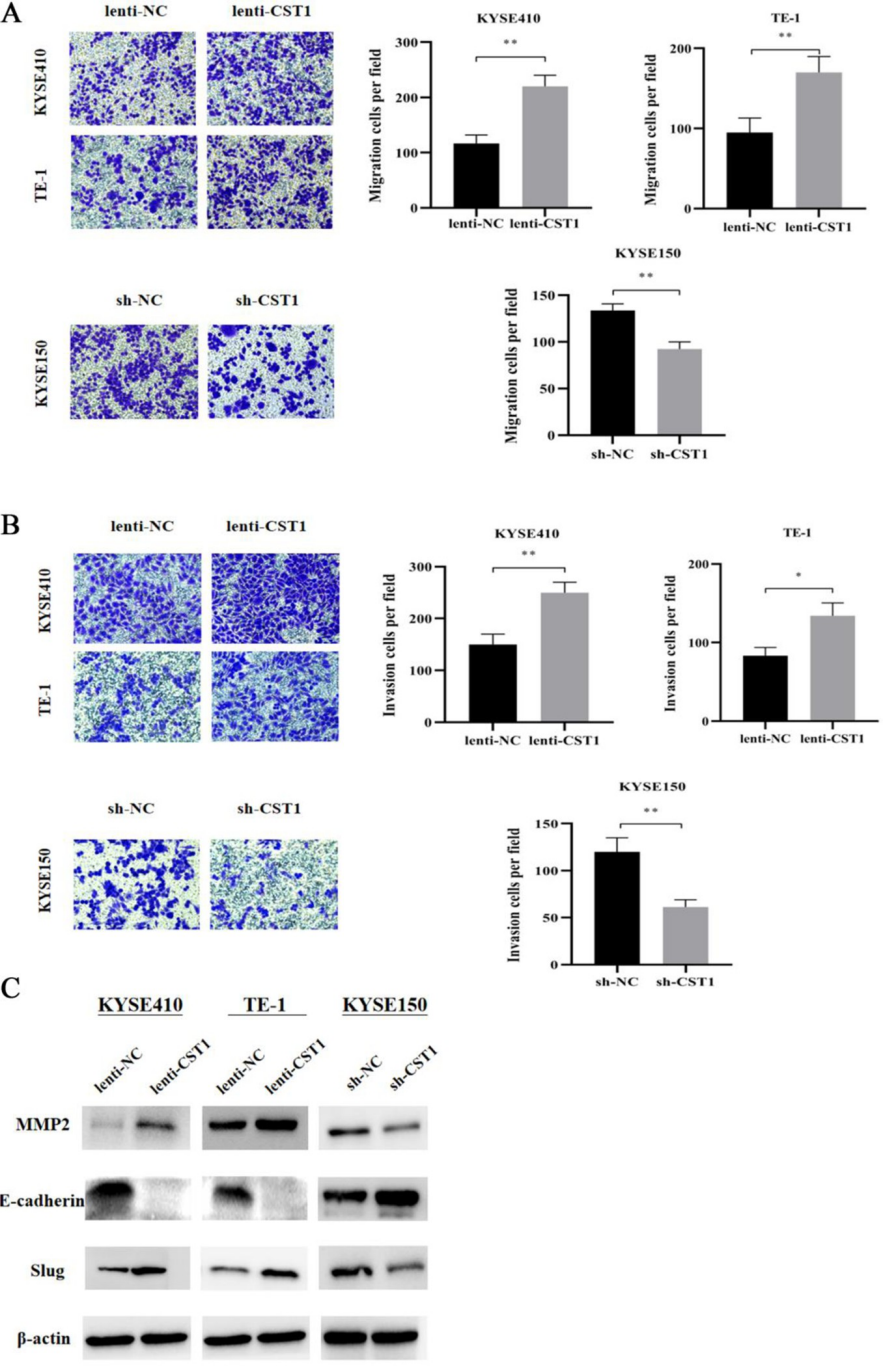
Effect of CST1 on migration and invasion as well as EMT-related protein expression of ESCC cells (×200). (A) CST1 overexpression/knockdown cell migration. (B) CST1 overexpression/knockdown cell invasion. (C) Expression of EMT-related proteins MMP2, E-cadherin and Slug.(* *P*<0.05, ** *P*<0.01, *** *P*<0.001, **** *P*<0.0001).

To investigate the role of CST1 on ESCC development, we examined the expression of CST1 in ESCC cells. Western blot results showed that CST1 expression was relatively high in KYSE150 and relatively low in the other six cell lines TE-1, TE-13, KYSE410, KYSE30, ECA109, KYSE140 expression (Fig 3A). Subsequently, we constructed two CST1 overexpression cells, KYSE410 and TE-1, and one CST1 knockdown cell KYSE150 (Fig 3B). Transwell assay results showed that, compared with the vector control, CST1 overexpression promoted the migration and invasion of KYSE410 and TE-1 cells, while CST1 knockdown inhibited the migration and invasion of KYSE150 cells (Fig 4A and 4B). In addition, we determined the effect of CST1 on epithelial-mesenchymal transition (EMT) in ESCC cells. Western blot results showed that CST1 overexpression promoted the expression of EMT-related transcription factor Slug, matrix metalloproteinase MMP2, and inhibited the expression of epithelial marker E-cadherin; while CST1 knockdown inhibited the expression of Slug, MMP2, and promoted the expression of E-cadherin (Fig 4C). These results indicated that CST1 might enhance the migration and invasion ability by promoting the EMT process of ESCC cells.

### CST1 promotes migration and invasion of ESCC cells through MEK/ERK/ CREB pathway

**Fig 5:**
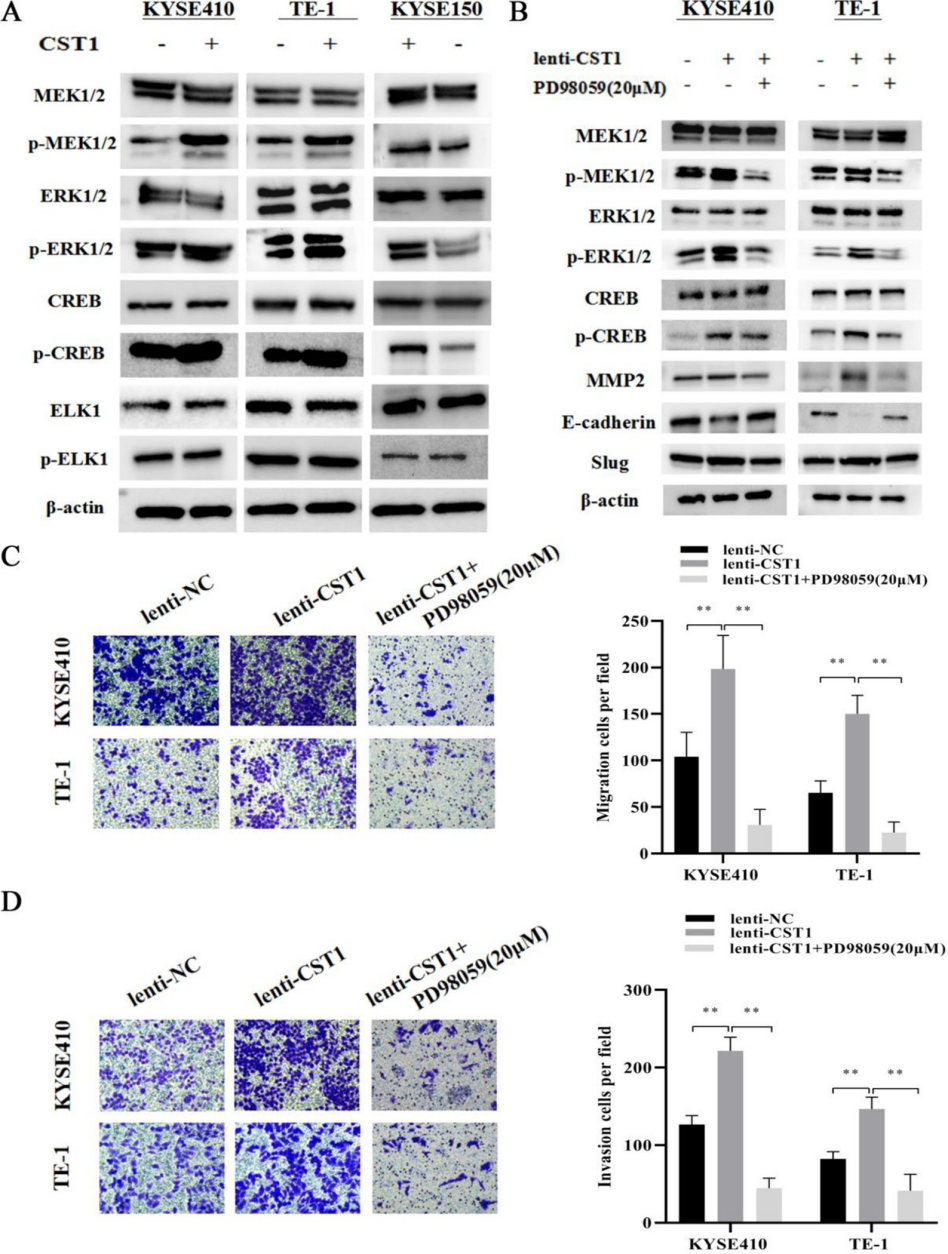
Effect of CST1 on MEK/ERK/CREB pathway. (A) Changes in MEK/ERK/CREB pathway proteins. (B) Changes in MEK/ERK/CREB pathway and EMT-related proteins after the addition of inhibitor PD98059 (20μM). (C) Changes in cell migration ability after the addition of inhibitor PD98059 (×200). (D) Changes in cell invasion ability after the addition of inhibitor PD98059 (×200). (** *P*<0.01).

MEK/ERK pathway has been demonstrated to be responsible for the EMT process of malignancies [17, 18], therefore, we examined the effect of CST1 on the phosphorylation of MEK/ERK and the key downstream effectors including CREB and ELK1. Western blot results showed that CST1 overexpression upregulated the expression of p-MEK1/2, p-ERK1/2, and p-CREB, while CST1 knockdown downregulated the expression of p-MEK1/2, p-ERK1/2, and p-CREB (Fig 5A). However, the phosphorylated level of ELK1, another key downstream effector of the MEK/ERK, was not significantly changed by CST1 overexpression/knockdown. To further investigate whether the activation of MEK/ERK/CREB pathway is involved in the EMT process of ESCC cells, we used PD98059 to inhibit the phosphorylation of MEK/ERK and then observed the subsequent changes of EMT proteins and cell migration and invasion ability. Western blot results showed that the expression of Slug, MMP2 was downregulated, whereas the expression of E-cadherin was upregulated (Fig 5B). Transwell assay results showed that the addition of PD98059 (20 μM) inhibited the ability of CST1 to promote the migration and invasion of ESCC cells (Fig 5C and 5D). These results indicated that CST1 might promote ESCC migration and invasion by MEK/ERK/CREB pathway.

### MiR-942-5p inhibits the migration and invasion of ESCC cells by regulating the expression of CST1

**Fig 6:**
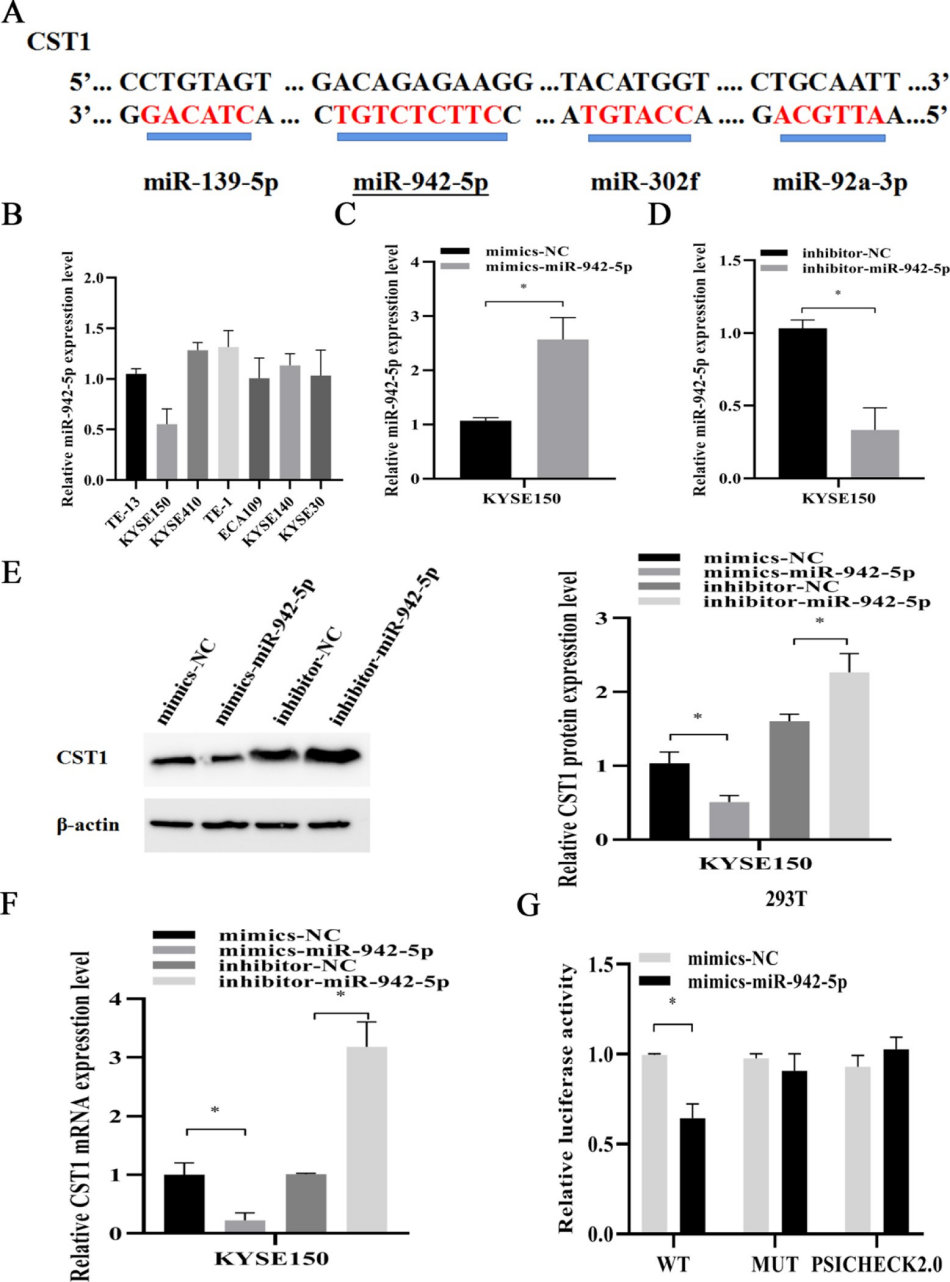
MiR-942-5p regulates the expression of CST1. (A) Conserved binding sites of four miRNAs to CST1 sequences. (B) Expression of miR-942-5p in 7 cell lines. (C) Expression of miR-942-5p after transfection of mimics. (D) Expression of miR-942-5p after transfection of inhibitor. (E) Effect of miR-942-5p on CST1 protein levels. (F) Effect of miR-942-5p on CST1 mRNA levels. (G) Dual luciferase assay demonstrated the regulation of CST1 by miR-942-5p. (*P<0.05, ** *P*<0.01).

**Fig 7:**
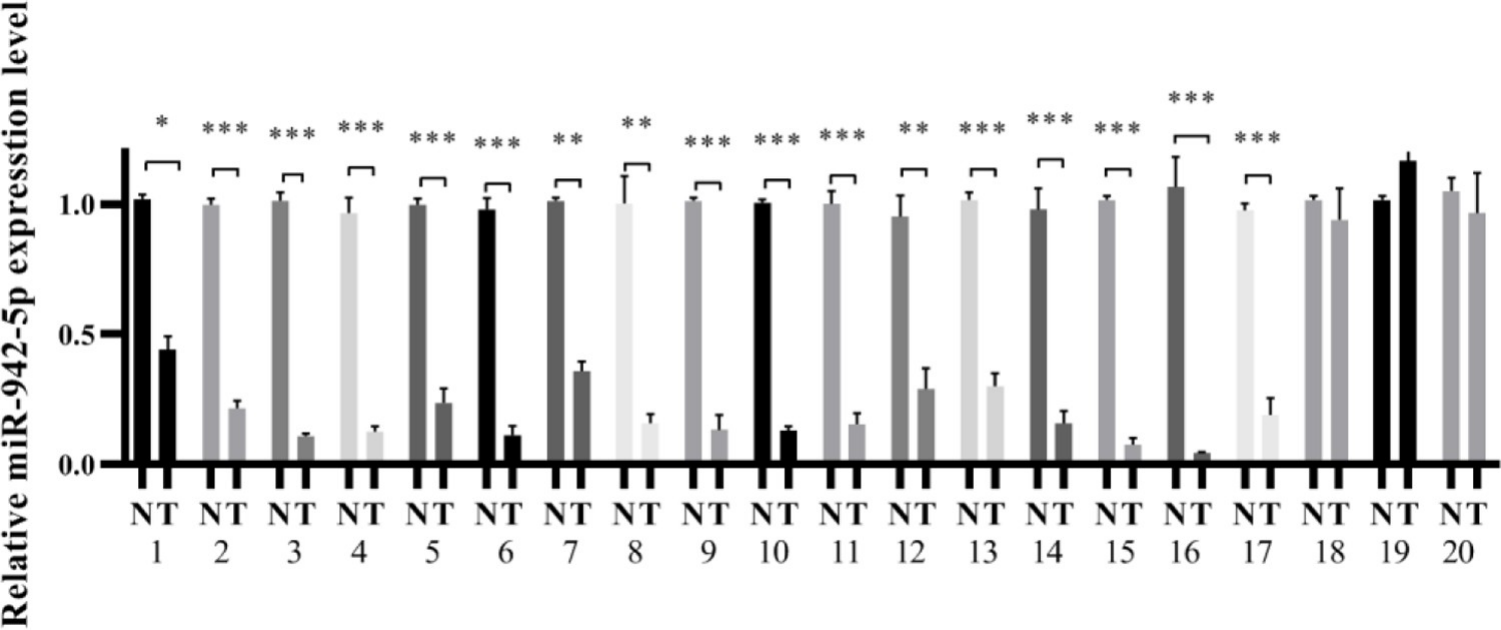
The expression of miR-942-5p in cancerous and paired paracancerous tissues of ESCC patients. (*P<0.05, ** *P*<0.01,*** *P*<0.01).

**Fig 8:**
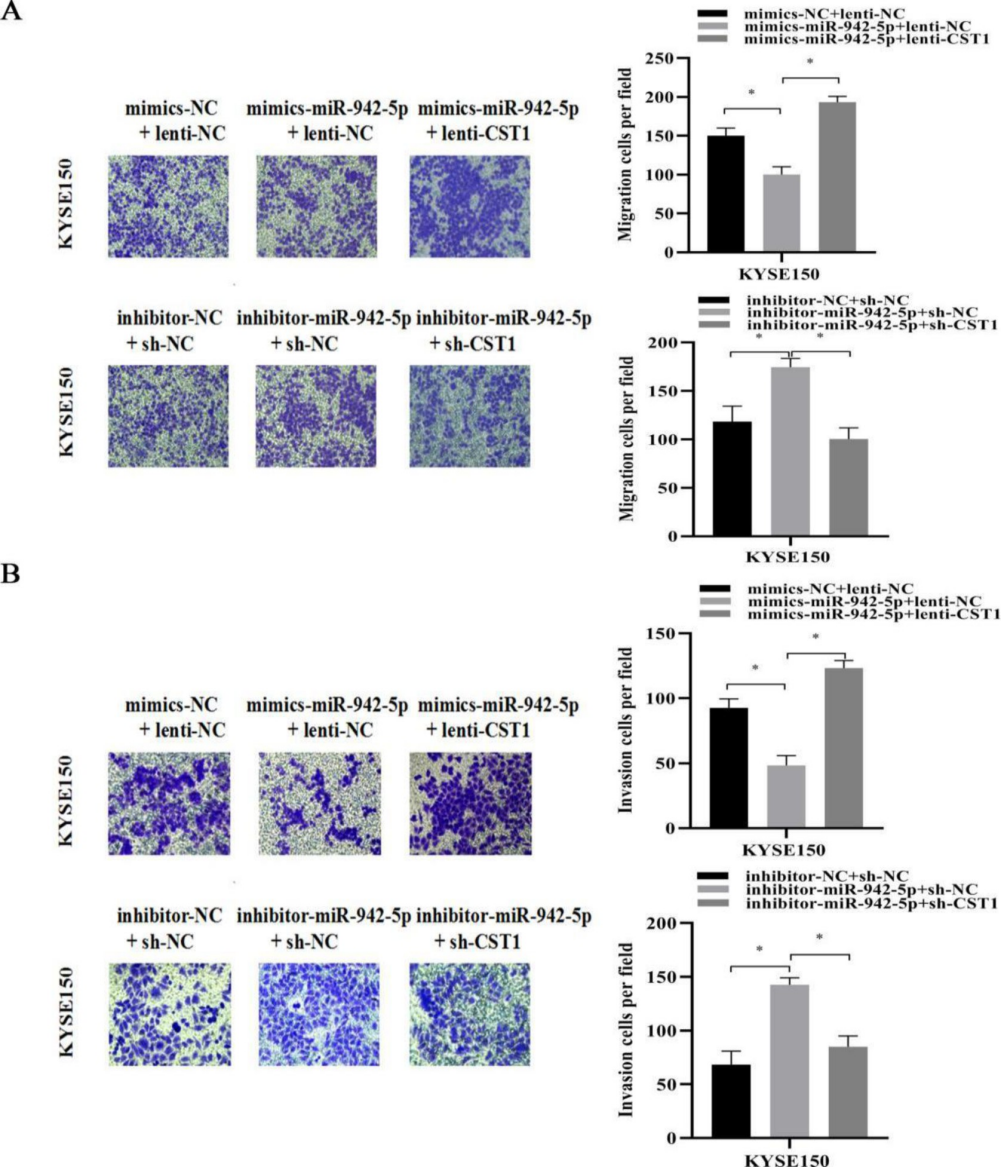
Effect of miR-942-5p on migration and invasion of ESCC cells (×200). (A) MiR-942-5p inhibits migration in KYSE150 cells. (B) MiR-942-5p inhibits invasion in KYSE150 cells. (* *P*<0.05).

To explore the upstream miRNAs of CST1, we screened four miRNAs including hsa-miR-942-5p, hsa-miR-139-5p, hsa-miR-92a-3p, and hsa-miR -302f predicted to target the CST1 gene through three databases, TargetScan, microRNA.ORG, and miRDB, and then the conserved binding sites of four miRNAs for CST1 were detected by Snapgene software. Among them, the hsa-miR-942-5p sequence had binding sites with CST1 reaching 7mer-m8, and the rest only 6mer (Fig 6A), so we focused on exploring the effect of miR-942-5p on CST1. Firstly, we examined the basal expression of miR-942-5p in seven ESCC cells, and the results showed that KYSE150, a cell with relatively high CST1 expression, had lower miR-942-5p expression than other cells (Fig 6B). Then, we added different mimics and inhibitor to the KYSE150 cell medium. RT-qPCR and western blot results showed that miR-942-5p mimics downregulated the expression of CST1 mRNA level and protein level, while miR-942-5p inhibitor upregulated the expression of CST1 mRNA and protein (Fig 6C–6F); the dual-luciferase assay further confirmed the regulatory effect of miR-942-5p on CST1 expression (Fig 6G), while the other three miRNAs did not affect on CST1 expression. Moreover, by RT-qPCR of 20 pairs of cancerous tissues and paracancerous tissues of ESCC patients, we also found that the miR-942-5p levels in cancerous tissues were significantly lower than those in paracancerous tissues, which was contrary to the high CST1 expression in cancerous tissues, suggesting that miR-942-5p may have a regulatory effect on CST1 (Fig 7). Lastly, transwell assay results showed that, compared with the control, the overexpression of miR-942-5p inhibited the migration and invasion ability of KYSE150 cells, and this effect was reversed by the overexpression of CST1, whereas the knockdown of miR-942-5p promoted the migration and invasion of KYSE150 cells and was reversed by the downexpression of CST1 (Fig 8A and 8B). The above results indicated that miR-942-5p might inhibit the ESCC migration and invasion by regulating the expression of CST1.

## Discussion

Recent studies have indicated that CST1 might play the potential carcinogenic role on some malignancies [4–10]. For example, Liu et al [6] reported that CST1 was involved in the development of ER+ breast cancer by regulating the ERα/ PI3K/Akt /ERα loop. For hepatocellular carcinomas, CST1 was found to promote the process of EMT in tumor cells by regulating the PI3K/Akt pathway [9]. Additionally, CST1 expression was demonstrated to be responsible for the elevation of migration and invasion ability of gastric cancer cells by regulating the Wnt pathway [10]. However, the role of CST1 on ESCC has not yet been clarified up to now. Interestingly, our pilot study suggested that CST1 might be involved in the tumorigenesis of ESCC manifested with ectopic high expression of CST1 in both sera and cancerous tissues of the patients with ESCC [12] Aberrant CST1 expression existed in ESCC was further profiled in this study. First, by TCGA database CST1 expression was shown significantly upregulated in esophageal cancer tissues, and closely correlated with grades, stages, lymph node metastasis and survival probability; And then, by immunohistochemistry staining, CST1 protein was shown aberrantly expressed in ESCC tissues with the positive rate up to 68.1% (32/47) in cancerous tissues significantly higher than 0% (0/47) in paracancerous tissues (*P*<0.01), and closely correlated with tumor stages. Additionally, by RT-qPCR, CST1 mRNA has also exhibited markedly higher expression in cancerous tissues than that in paracancerous tissues. All the above findings strongly indicated that CST1 might play an important role in the development of ESCC.

To investigate whether aberrant CST1 expression is responsible for the ESCC development, we performed the evaluation of the effect of CST1 expression on ESCC cells by constructing CST1 overexpression/knockdown cells. As expected, CST1 presented the carcinogenic role on ESCC. It was shown that CST1 overexpression significantly upregulated not only the phosphorylation of key effectors MEK1/2 and ERK1/2 in MEK/ERK pathway, but also the expression of EMT-related molecular proteins including transcription factor Slug and matrix metalloproteinase MMP2, and inhibit the expression of epithelial marker E-cadherin, thus promoting the migration and invasion ability of ESCC cells. On the other hand, CST1 knockdown presented the opposite effect on ESCC cells compared with that of CST1 overexpression. Furthermore, by the addition of p-MEK1/2/p-ERK1/2 inhibitor PD98059, we demonstrated that the MEK / ERK signal inhibition is responsible for the inhibition of EMT- related proteins expression and ESCC cell migration and invasion. Especially mentioned, we determined the effect of CST1 on the phosphorylation of CREB other than ELK1 in ESCC cells, although either of which is the key downstream effector of MEK/ERK, and the MEK/ERK/CREB pathway has been demonstrated its role on regulating cell malignant biological behaviors as well as EMT-related process in many malignancies [19, 20] Therefore, it is reasonable to deduce that CST1 might promote the migration and invasion of ESCC cells by activating MEK/ERK/CREB pathway.

Finally, to define the upstream miRNAs regulating CST1 expression, we performed the screening of four miRNAs with the highest relevance to CST1 by three databases including TargetScan, microRNA.ORG and miRDB. By Snapgene software analysis, it was shown that MiR-942-5p had 7mer-m8 binding to the CST1 sequence sites, while the other three miRNAs had only 6mer. And then, by RT-qPCR, western blot, transwell assay and dual-luciferase reporter assay, we observed that miR-942-5p had an inhibitory effect on the migration and invasion ability of ESCC cells and the effect could be reversed by the expression of CST1. MiR-942-5p expression significantly decreased the expression of CST1, confirming the regulatory role of miR-942-5p on CST1.

In summary, to the best of our knowledge, this study is the first to unveil the carcinogenic role of CST1 on ESCC by activating the MEK/ERK/CREB pathway to promote cell migration and invasion, and the regulatory effect of miR-942-5p on targeting CST1, suggesting that miR-942-5p/CST1 axis might be a promising target for diagnosis and treatment of ESCC. Moreover, it is still essential further studies should be carried out to determine whether other signaling pathways also participate in miR-942-5p/CST1 axis mediated carcinogenesis in ESCC.

## Supporting information

S1 Raw images (PDF)
