# Supplementary figures and images for: MiR-942-5p inhibits tumor migration and invasion through targeting CST1 in esophageal squamous cell carcinoma
Source: PLoS One. 2023 Feb 27;18(2):e0277006. doi: 10.1371/journal.pone.0277006 (PMC9970063; doi:10.1371/journal.pone.0277006)

Fig 3A

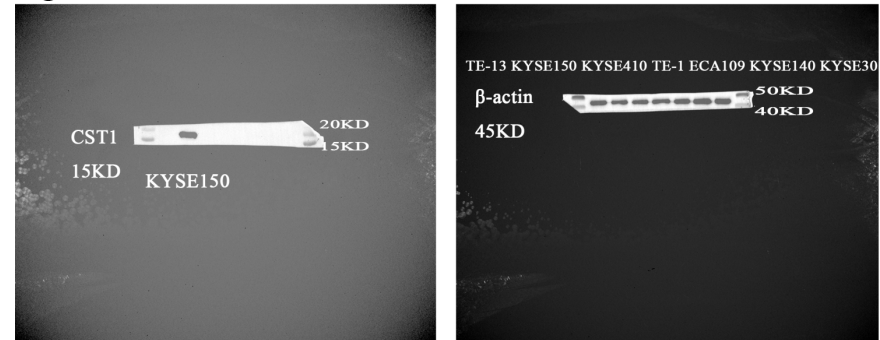

Fig 3B

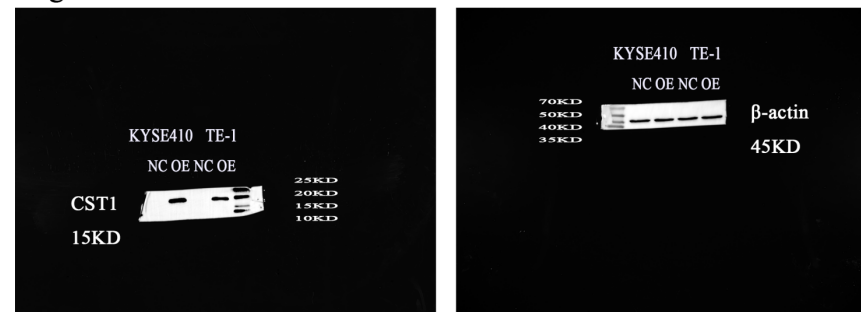

Fig 3B

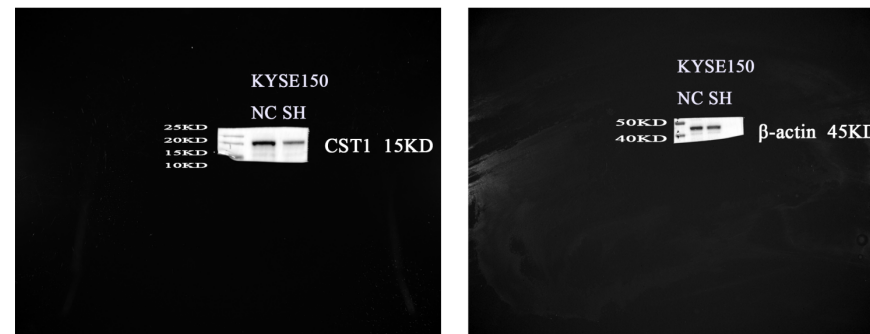

Fig 4C

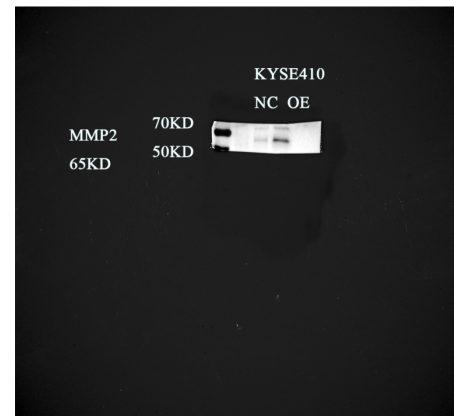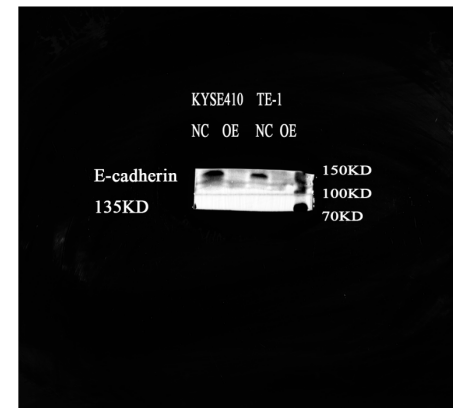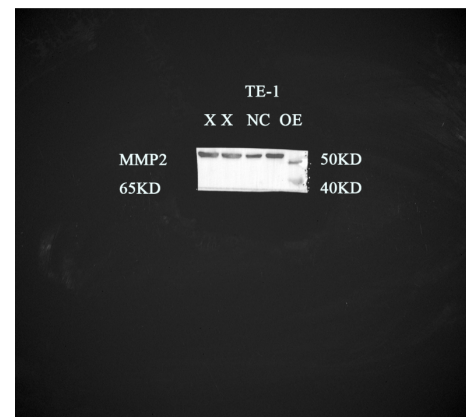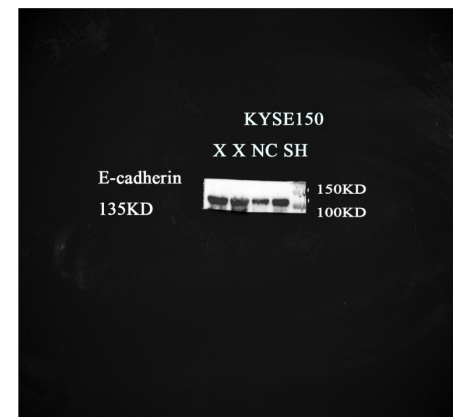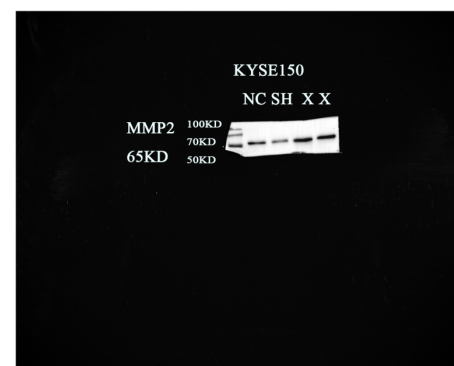

Fig 4C

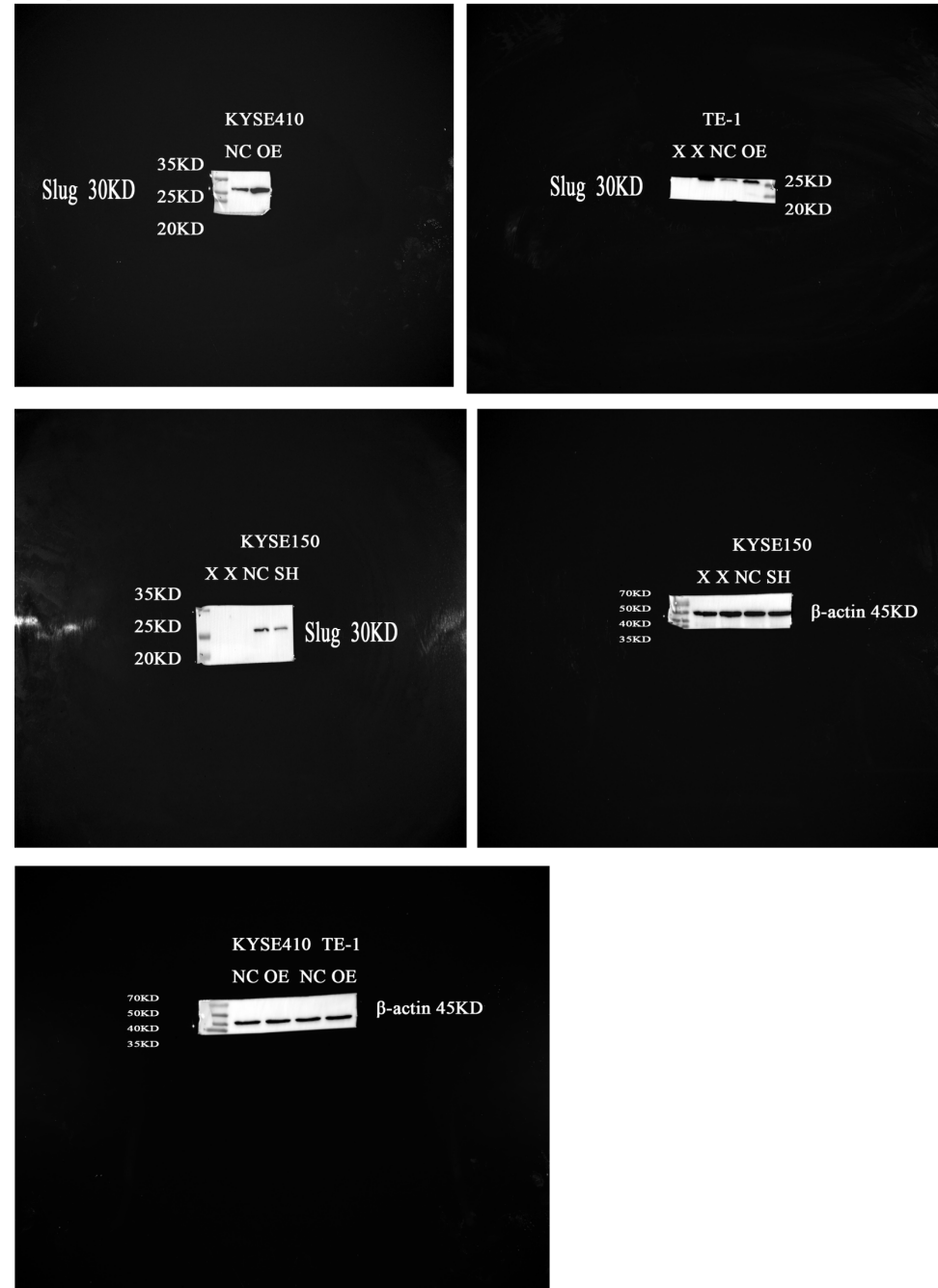

Fig 5A

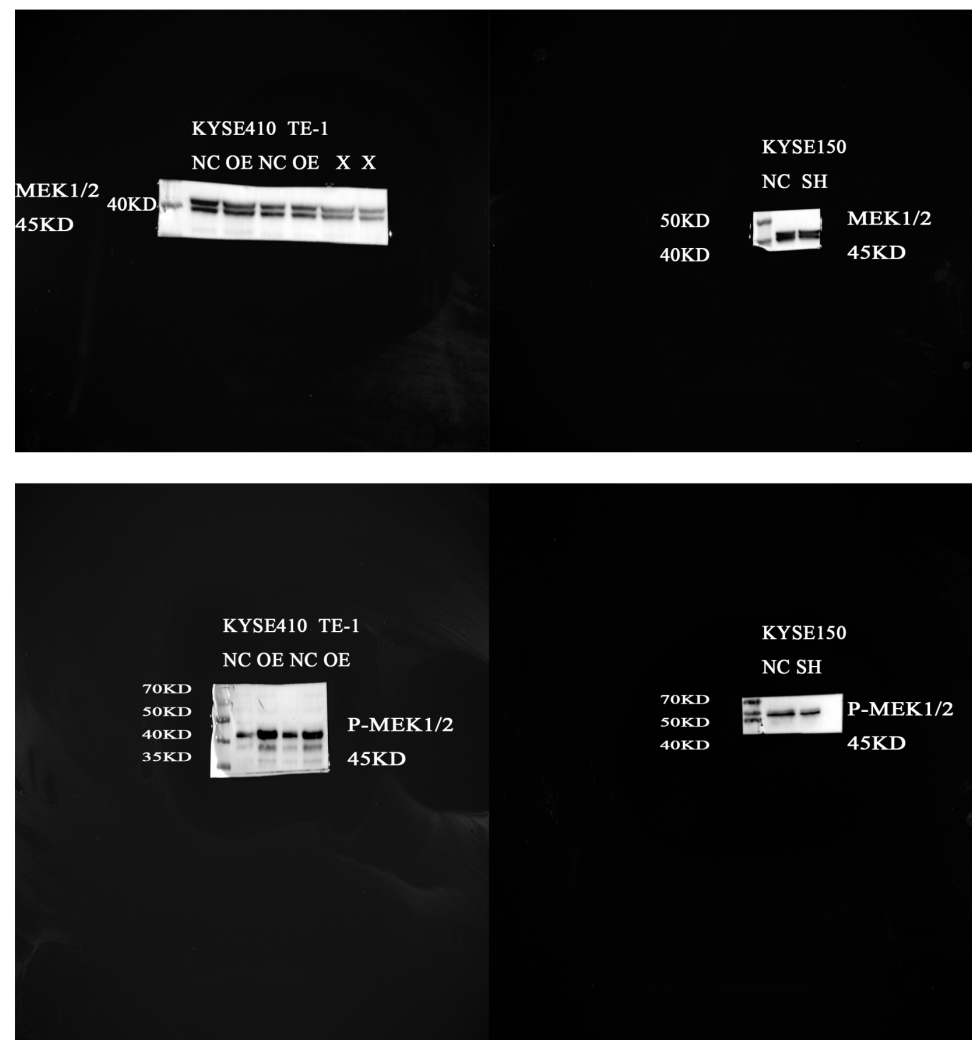

Fig 5A

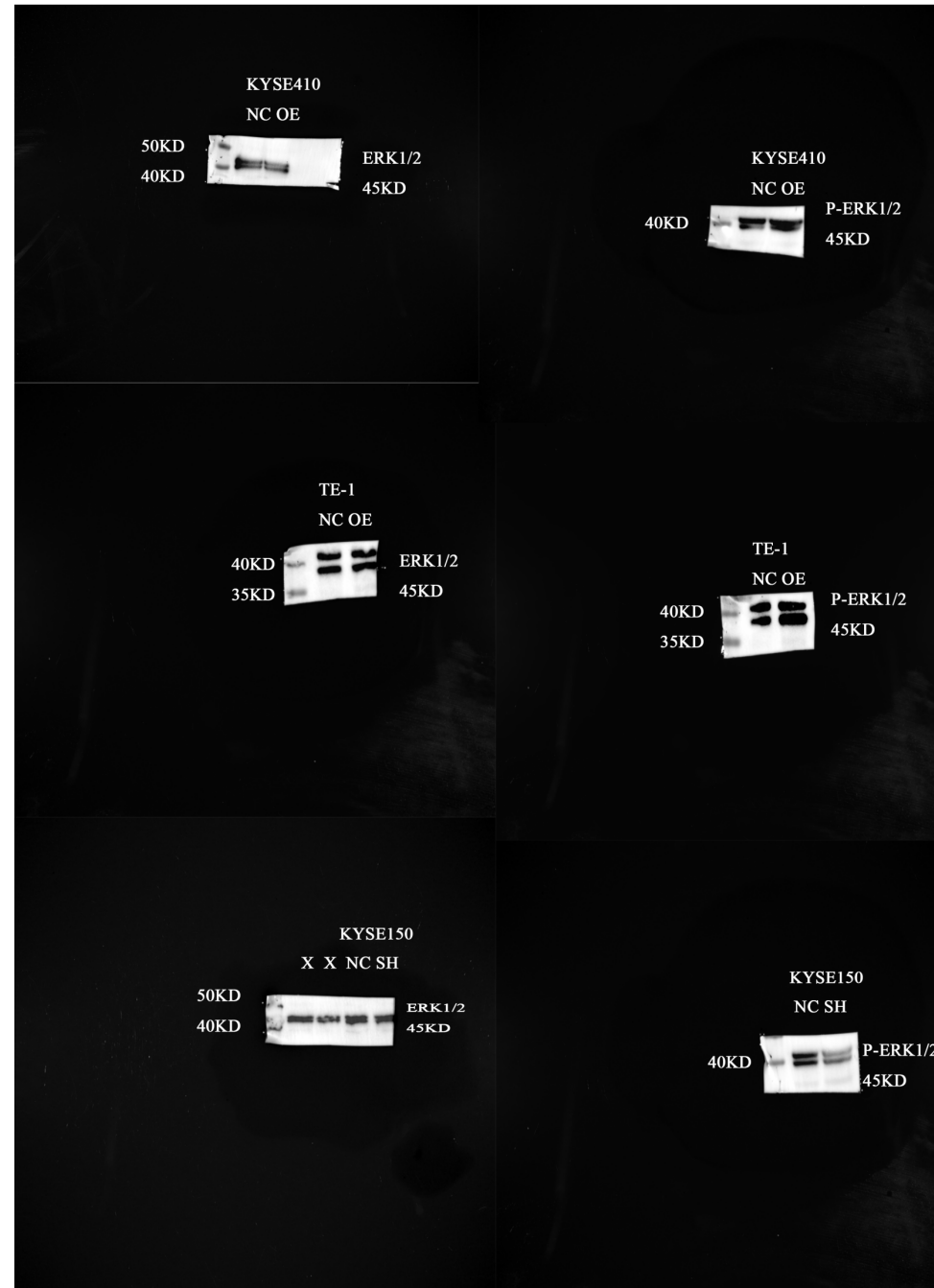

Fig 5A

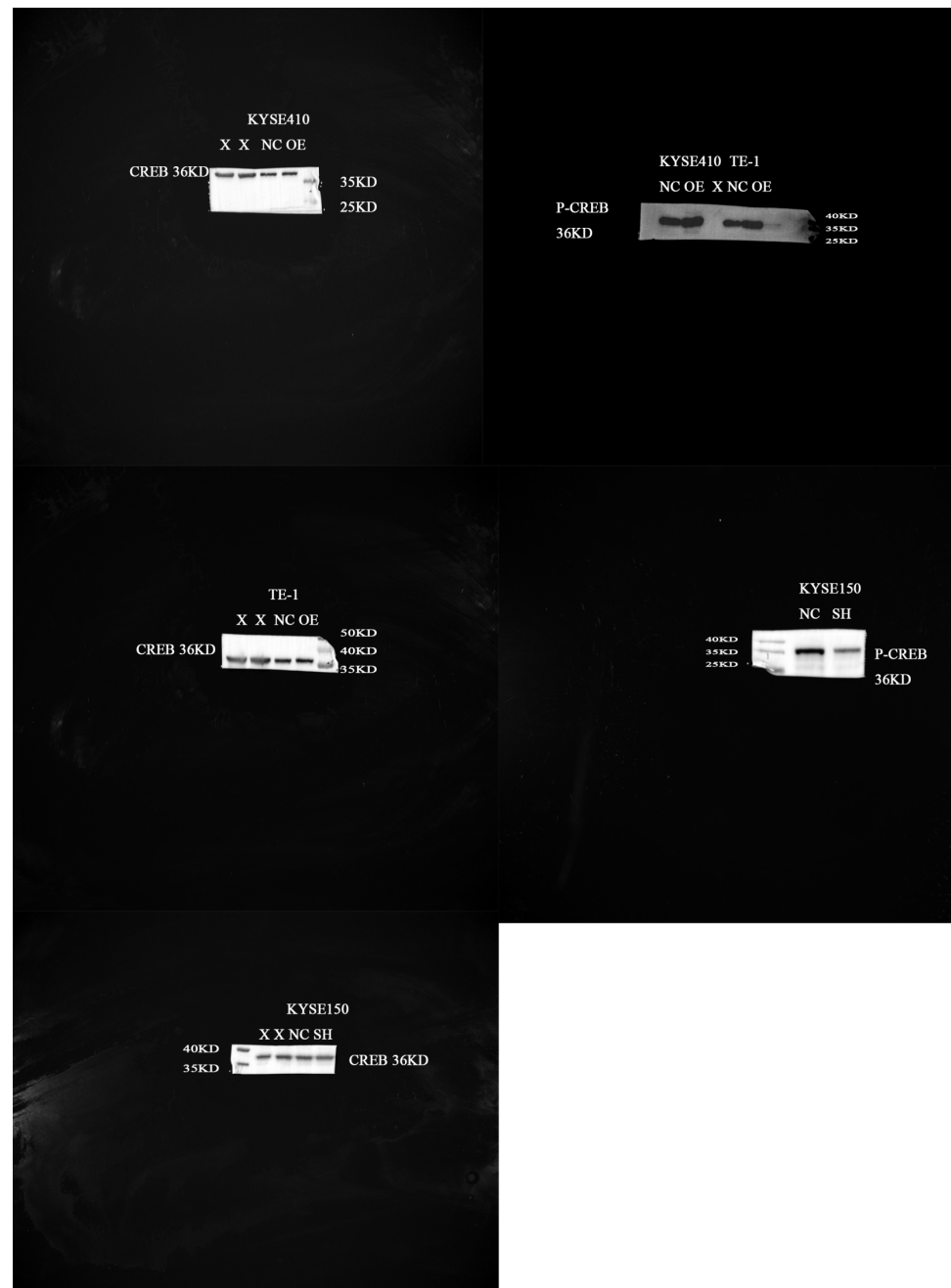

Fig 5A

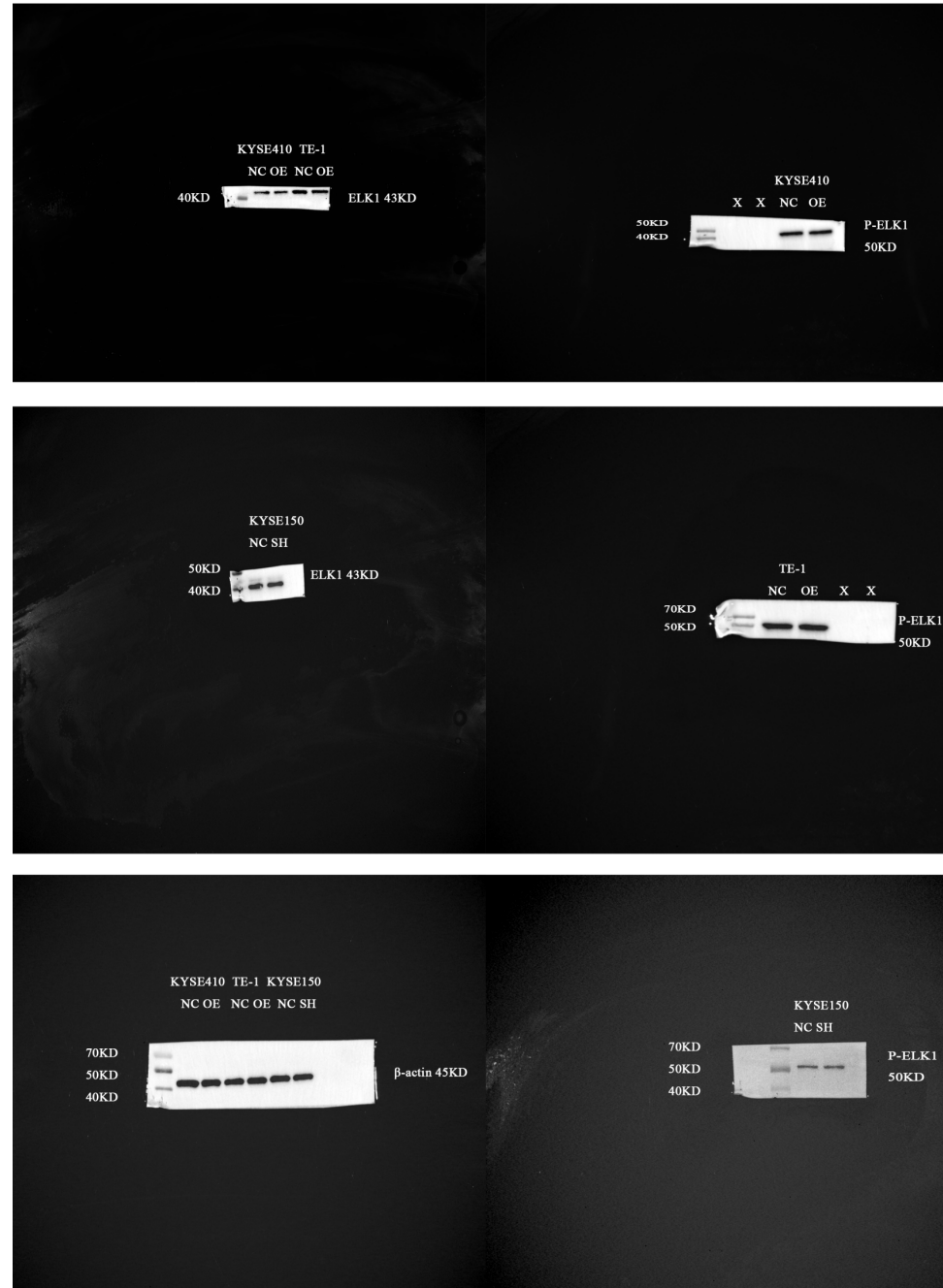

Fig 5B

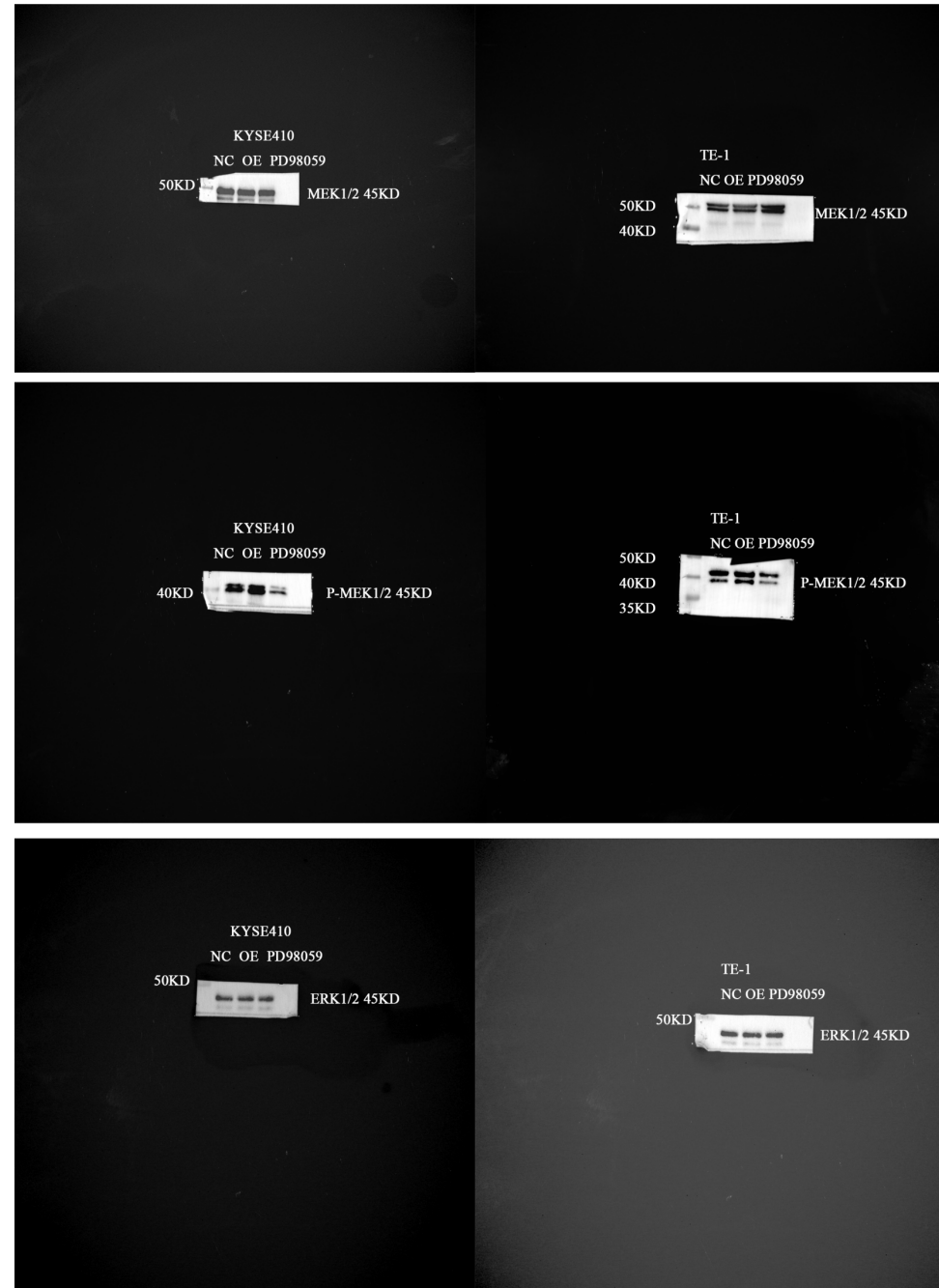

Fig 5B

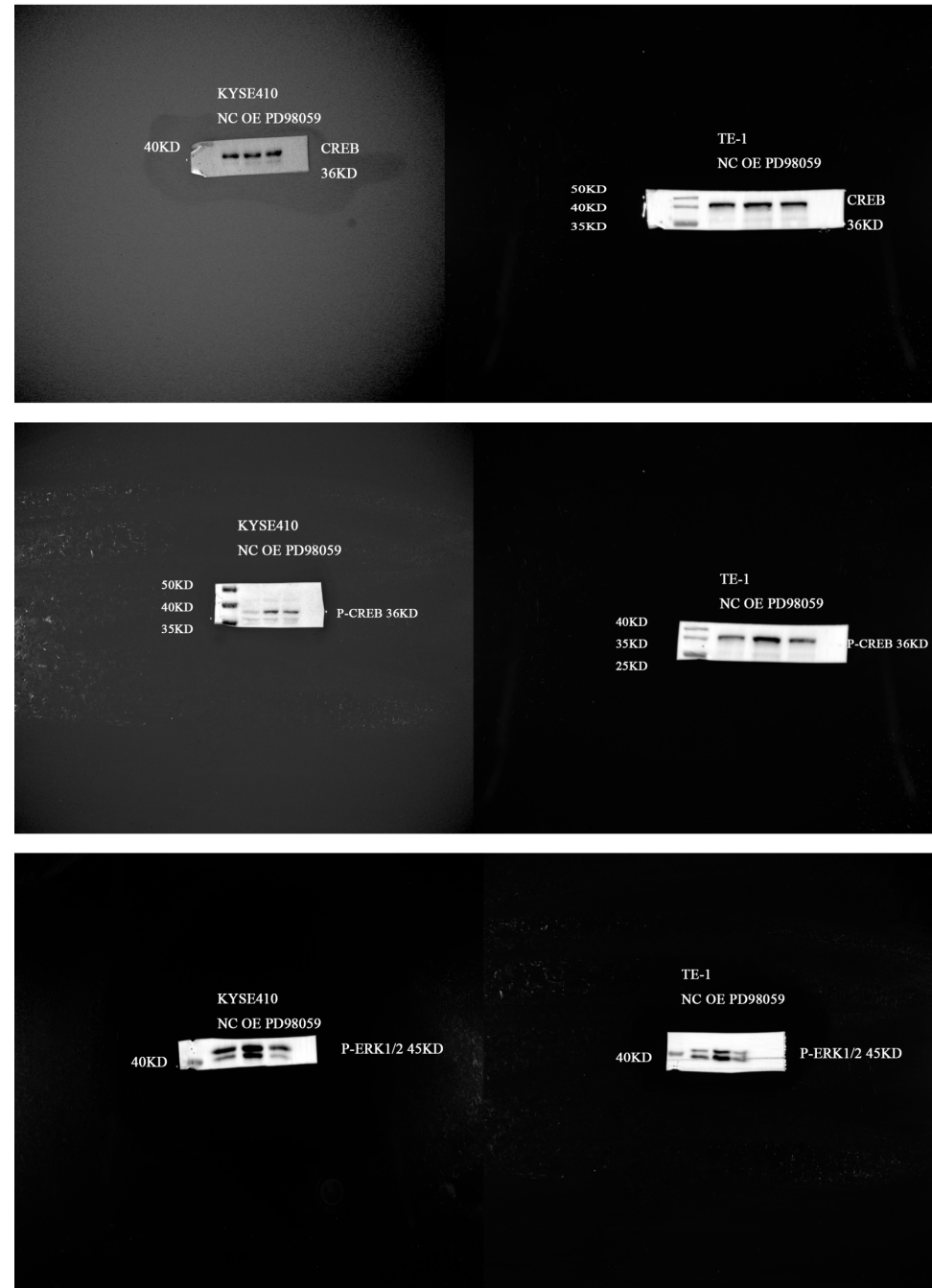

Fig 5B

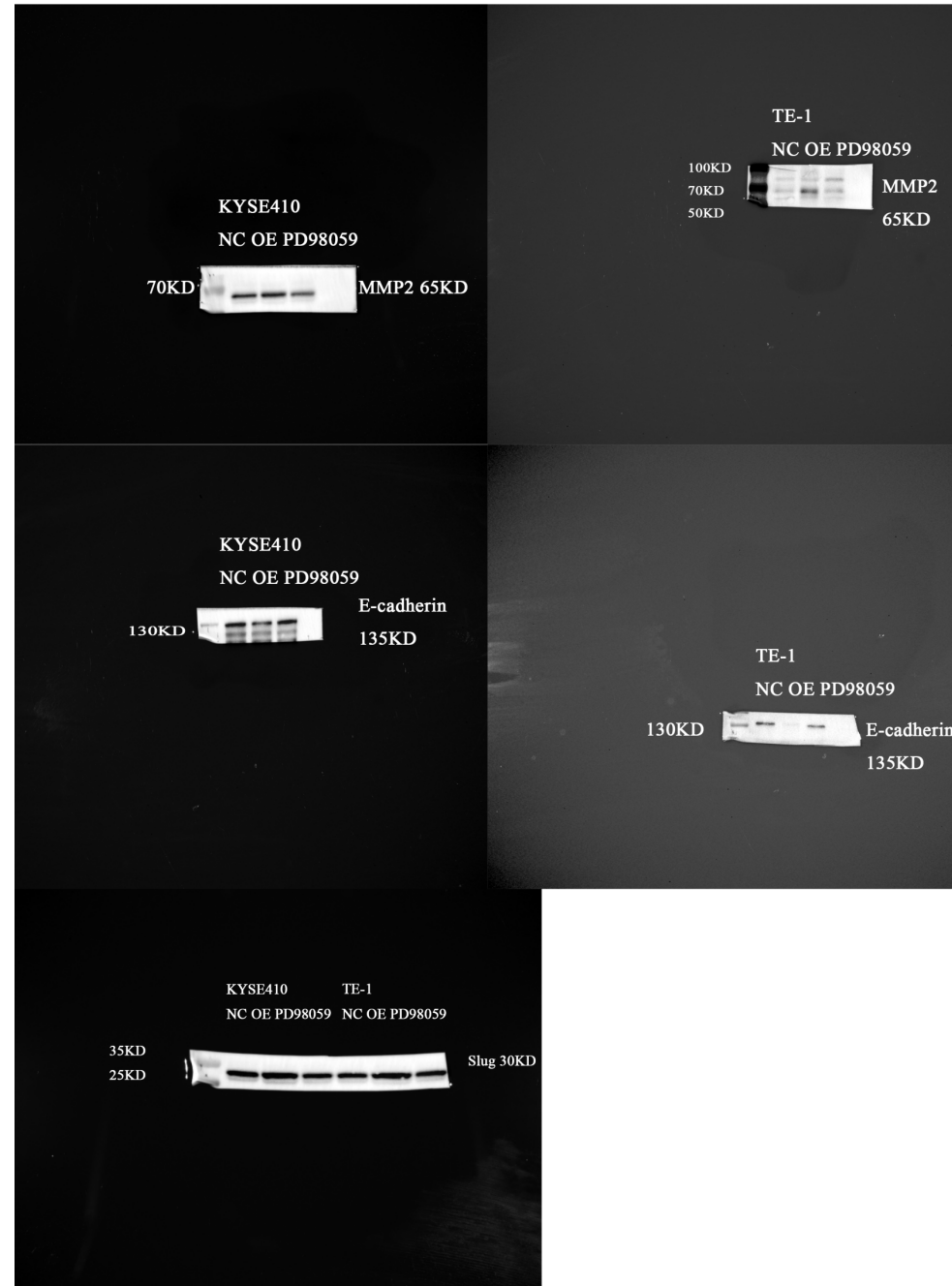

Fig 5B

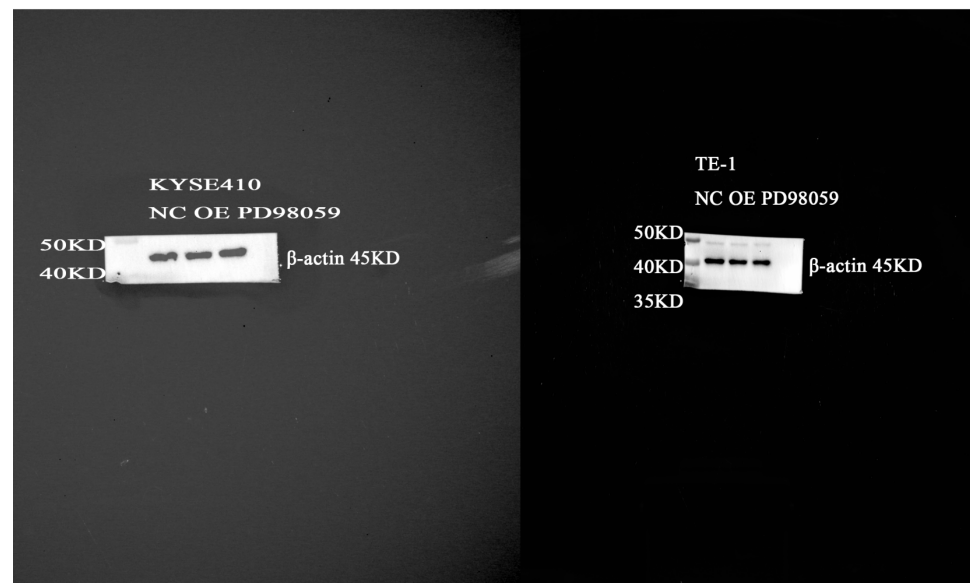

Fig 6E

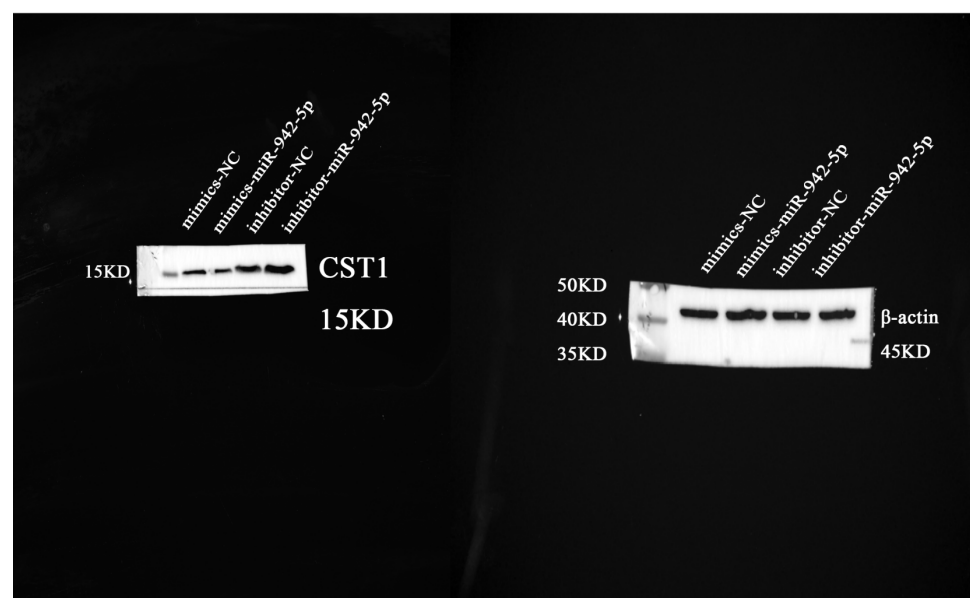

Supplement: S1 Raw images — (PDF) [file pone.0277006.s001.pdf]
